# Effects of Daily Iron Supplementation on Motor Development and Brain Connectivity in Preterm Infants: A Diffusion Magnetic Resonance Study

**DOI:** 10.3389/fnins.2021.769558

**Published:** 2021-11-08

**Authors:** Mingyan Li, Chai Ji, Weifeng Xuan, Weijun Chen, Ying Lv, Tingting Liu, Yuqing You, Fusheng Gao, Quan Zheng, Jie Shao

**Affiliations:** ^1^Department of Child Health Care, National Clinical Research Center for Child Health, The Children’s Hospital, Zhejiang University School of Medicine, Hangzhou, China; ^2^Shaoxing Maternal and Child Health Care Hospital, Shaoxing, China; ^3^Key Laboratory for Biomedical Engineering of Ministry of Education, Department of Biomedical Engineering, College of Biomedical Engineering and Instrument Science, Zhejiang University, Hangzhou, China; ^4^Department of Radiology, National Clinical Research Center for Child Health, Children’s Hospital, Zhejiang University School of Medicine, Hangzhou, China

**Keywords:** preterm infant, iron supplementation, motor development, brain structural connectivity, diffusion MRI

## Abstract

**Objectives:** The aim of the study is to demonstrate the characteristic of motor development and MRI changes of related brain regions in preterm infants with different iron statuses and to determine whether the daily iron supplementation can promote motor development for preterm in early infancy.

**Methods:** The 63 preterm infants were grouped into non-anemia with higher serum ferritin (NA-HF) group and anemia with lower serum ferritin (A-LF) group according to their lowest serum Hb level in the neonatal period as well as the sFer at 3 months old. Forty-nine participants underwent MRI scans and Infant Neurological International Battery (INFANIB) at their 3 months. At 6 months of corrected age, these infants received the assessment of Peabody Developmental Motor Scales (PDMS) after 2 mg/kg/day iron supplementation.

**Results:** In total, 19 preterm infants were assigned to the NA-HF group while 44 preterm infants to the A-LF groups. The serum ferritin (sFer) level of the infants in A-LF group was lower than that in NA-HF group (44.0 ± 2.8 mg/L vs. 65.1 ± 2.8 mg/L, *p* < 0.05) and was with poorer scores of INFANIB (66.8 ± 0.9 vs. 64.4 ± 0.6, *p* < 0.05) at 3 months old. The structural connectivity between cerebellum and ipsilateral thalamus in the NA-HF group was significantly stronger than that in the A-LF group (*n* = 17, 109.76 ± 23.8 vs. *n* = 32, 70.4 ± 6.6, *p* < 0.05). The decreased brain structural connectivity was positively associated with the scores of PDMS (*r* = 0.347, *p* < 0.05). After 6 months of routine iron supplementation, no difference in Hb, MCV, MCHC, RDW, and sFer was detected between A-LF and NA-HF groups as well as the motor scores of PDMS-2 assessments.

**Conclusion:** Iron status at early postnatal period of preterm infant is related to motor development and the enrichment of brain structural connectivity. The decrease in brain structural connectivity is related to the motor delay. After supplying 2 mg/kg of iron per day for 6 months, the differences in the iron status and motor ability between the A-LF and NA-HF groups were eliminated.

## Introduction

Iron deficiency (ID) continues to be the most prevalent nutrient deficiency in the world. As we know, preterm infants are more likely susceptible to be anemic than term infants, most of which were due to ID (Todorich et al., 2009). Iron is an essential mineral necessary for delivering oxygen to tissues throughout the body as well as serving important roles in metabolism, respiration, and immune functions (Todorich et al., 2009). It is also a cofactor in the central nervous system development processes (DellaValle, 2013). Our body carefully sustains a balance between iron loss, iron absorption, and iron storage. ID is a precursor to iron deficiency anemia (IDA). The first stage of ID is characterized by a decrease in serum ferritin (sFer), which is caused by the depletion of total body iron stores, while other iron indices and hemoglobin (Hb) remain normal. The IDA stage is not only symbolized by lower sFer and hemoglobin but also by lower mean corpuscular volume (MCV), lower mean hemoglobin concentration (MCHC), and higher red blood cell distribution width (RDW).

Brain ID occurs before IDA. It can alter the development of oligodendrocytes and result in hypomyelination of white matter, which is related to changes in startle response, auditory evoked potentials, and motor function in the infant (Beard, 2007; Todorich et al., 2009). Early ID also neurochemically alters the function of neurotransmitters. Animal models have shown that ID can alter the function of the frontal cortex, midline thalamus, and other brain regions by modifying the dopaminergic neurotransmission system (Beard and Connor, 2003).

Brain ID in fetuses or neonates is more detrimental than in toddler because of the rapidity of brain growth early in life. Obtaining adequate iron of the developing brain is necessary for behavioral and motor development (Felt and Lozoff, 1996). A number of studies have reported that term infants with IDA or chronic severe ID have lower motor development scores, compared with infants with normal iron status (Shafir et al., 2008). It is particularly concerning that the ID infants have poorer motor function because ID without anemia is more common than IDA, which cannot be detected by regular screening procedures (Shafir et al., 2008).

Compared with full-term infants, preterm infants are deprived of iron accretion that occurs in the third trimester of pregnancy, which results in a decrease in iron storage at birth, as reflected in the decrease in sFer (Lackmann et al., 1998; McCarthy et al., 2017). In addition, most studies have found that reduced brain iron concentration was accompanied with lower sFer (Georgieff, 2017). Therefore, sFer is a valuable index indicating the brain ID of these infants who are more susceptible to motor delay. As iron supplementation after birth could improve gross motor remarkably in term infants (Shafir et al., 2008), we speculate that early regular iron supplementation to these premature infants at high risk of ID can be beneficial to their motor development.

Clinical neuroimaging research on early motor abilities is still limited. Previous studies have found that structural connections between motor-related brain regions play an important role in movement development (Craig et al., 2020). Cranial magnetic resonance (MRI) is a common and valuable method to study the infant brain functions, and diffusion tensor imaging (DTI) is a non-invasive method to study the white matter of the brain. Great progress has also been made in the study of human brain architecture with ID or IDA by DTI and structural MRI (Hannah and D’Cruz, 2019). Probabilistic fiber tracking by DTI in ID adult has found that iron concentration is linked to structural connectivity of the subthalamic nucleus (Dimov et al., 2019). Therefore, MR tractography in premature infants with ID or IDA was a recommended method for exploring development delay.

To characterize the motor development and MRI changes of related brain regions in preterm infants with different iron status, we examined data from a follow-up study including brain imaging and behavior development of preterm infants with different levels of iron metabolism. The relationship between scores of gross and fine motor function of preterm infants and structural brain network based on DTI were analyzed. We hypothesized that preterm infants with neonatal anemia who have lower iron levels would present an altered brain network connectivity and motor ability. Routine iron supplementation can improve iron status as well as motor development.

## Materials and Methods

The present study was approved by the ethics committee of the Children’s Hospital, Zhejiang University School of Medicine (Permit Number: 2019-IRB-027). Parents who accepted the participation provided written informed consent before enrolled on the study. All the data used in the present study were available to the community *via* a suitable open repository.

### Participants

We conducted a follow up study of preterm infants who attended the High Risk Infant Clinic of the Children’s Hospital, Zhejiang University School of Medicine, from January 2018 to December 2019. Participants included 63 preterm infants, with a gestational age (GA) of 28–36 weeks. All the enrolled infants were invited for regular follow-up every month since 40 weeks GA. Information of medical records, including weight at birth, GA, neonatal complications, Hb during neonate period (the lowest Hb tested within the first month after birth), as well as anthropometric measurements were collected. The enrollment criteria included no chromosomal and genetic anomalies, no craniofacial anomalies, no neurological complications, no hematological disease, and no blood transfusions in the first 6 months (corrected age) of life.

### Iron Status Detection and Iron Supplementation

Venous blood samples of enrolled participants have been tested for estimation Hb and ferritin at 3 months, and sFer, Hb, MCV, MCHC, and RDW were collected at 6 months (correct age). Anemia was defined as venous Hb < 145 g/L within 28 days of age, Hb < 90 g/L at 3 months old, and < 110 g/L at 6 months old (Domellof, 2017). According to the lowest Hb level during the neonatal period as well as the sFer at 3 months, preterm infants were divided into two groups: anemia with lower ferritin group (A-LF, with Hb < 145 g/L) and non-anemia with higher ferritin group (NA-HF, with Hb ≥ 145 g/L). C-reactive protein (CRP) were all < 8 mg/L both at 3- and 6-month of age, which stands for no inflammation.

All participants in this study had delayed cord clamping at birth and were given iron supplements with a dosage of 2 mg/kg per day from 40 weeks of corrected GA according to the post-discharge feeding recommendations for premature, low birth weight infants in China (preterm infants in this study cohort were not introduced to have complementary food, so the total daily iron intake was calculated as the sum of iron supplements and the iron from formula and/or breast milk fortifier) (Wang and Liu, 2016).

### The Motor Ability Assessment

Early neurological function was evaluated according to the Infant Neurological International Battery (INFANIB) at 3 months of age. Results are expressed as raw scores for total motor ability. Peabody Developmental Motor Scales, second edition (PDMS-2), was conducted at 6 months of corrected age. The scales contain sub-tests of the following six parameters: (a) reflexes, (b) stationary (body control and equilibrium), (c) locomotion, (d) object manipulation, and (e) grasping. Raw scores are converted into age-equivalent scores for each sub-test; motor quotient is calculated from the standard scores of five sub-tests of PDMS-2. The assessment of PDMS-2 was performed by two pediatricians who had no knowledge of the medical history of the infants.

### Image Acquisition

Infants were scanned after receiving 50 mg/kg of enema or oral chloral hydrate within 3 months old. The scans were performed on a Philips 3.0T Achieva system with standard eight-channel head coils. Two sequences were used in this study: (1) 3-D sagittal T2-weighted sequence echo time [(TE) = 278 ms, repetition time (TR) = 2,200 ms, acquisition matrix = 224 × 204, voxel size = 0.8 × 0.8 × 0.8 mm^3^, field of view (FOV) = 180 × 161 × 140 mm^3^]; (2) DTI images were collected using an echo-planar image (EPI) sequence with 32 non-colinear diffusion encoding directions for b value = 800 and 1,500 s/mm^2^ each, in addition with one non-weighted image (TE = 115 ms, TR = 9,652 ms, voxel size = 1.5 × 1.5 × 2 mm^3^, flip angle = 90°, FOV = 180 × 180 × 120 mm^3^, acquisition matrix = 120 × 118, bandwidth = 1,341 Hz/pixel, number of volumes = 60, 60 slices).

### Brain Region Segmentation and Volume Calculation Using T2-Weighted Imaging

The T2-weighted images were preprocessed including brain extraction (Smith, 2002), creation of brain mask, and bias correction (Tustison et al., 2010). Then the whole brain of each subject was segmented into 83 brain regions using Draw-EM (Developing brain Region Annotation With Expectation-Maximization) (Makropoulos et al., 2014, 2018), and the volume of each region was extracted.

### Diffusion Tensor Imaging Preprocessing

All DTI data were performed intra-subject registration using a linear image registration tool FLIRT (Jenkinson and Smith, 2001; Jenkinson et al., 2002), followed by eddy current correction using “topup” and “eddy” in FSL (Andersson and Sotiropoulos, 2016). Fractional anisotropy (FA) and mean/axial/radial diffusivity (MD/AD/RD) maps were generated from the diffusion tensor using the weighted linear least squares method (Basser et al., 1994). The individual images were transformed to the JHU-neonate single brain DWI atlas using a non-linear transformation of the multi-channel large deformation diffeomorphic metric mapping (LDDMM) (Miller et al., 1993; Djamanakova et al., 2013). Then the JHU-neonate parcellation map, which included 126 regions of interest (ROIs), was transformed to the individual native space. Registration of all subjects was checked.

### Diffusion Tensor Imaging-Based Connectivity Analysis

Tractography was performed using a fiber orientation distribution-based probabilistic fiber tracking algorithm in MRtrix3,^1^ with the whole-brain mask as the seed, and the following parameters were used: a cutoff of 0.05 min/max length of 10/250 mm, step size of 0.5 mm. An asymmetric connection matrix was generated for each subject from the whole brain tractography based on the JHU-neonate parcellation map, and the number of connection fibers between ipsilateral and contralateral motor-related regions, including frontal cortex, striatum, cerebellum, and thalamus, which were implicated in supporting early motor development (Todorich et al., 2009), was extracted.

### Statistical Analysis

All analyses were performed using SPSS software, version 16.0 (IBM Corporation, Armonk, NY, United States).

An independent sample *t*-test was performed on the iron metabolism parameters, age and birth weight. A Chi-square test was performed on gender and maternal education background.

ROIs were paired to compare the connectivity between A-LF and NA-HF groups. As all the data form a normal distribution and homogeneity, covariance analyses of the volume and connectivity of the brain regions, as well as the motor scores of PDMS-2, were performed by controlling GA and physical age before homogeneity test of variance. Multiple linear regression was used to test the relationship between INFANIB scores and the number of connection fibers/brain region volumes. Multiple comparison correction was conducted, and adjusted *p* < 0.05 was considered as statistically significant.

## Results

### Demographic and Clinical Information

A total of 63 infants were enrolled in the present study. Demographic and clinical information are shown in Table 1. GA and birth weight were lower in the A-LF group than in the NA-HF group. No difference was detected in gender, maternal age at delivery, and maternal education background between the two groups. The neonatal Hb level of the A-LF group was significantly lower than that of the NA-HF group (*n* = 44, Hb:114.9 ± 6.3 g/L vs. *n* = 19, Hb:174.1 ± 2.8 g/L, *p* < 0.05). Although there was no significant difference of Hb between the A-LF and NA-HF groups at 3 months old (*n* = 44, Hb: 102.1 ± 1.3 mg/L vs. *n* = 19, Hb: 97.6 ± 2.7 g/L, *p* = 0.127), sFer of the A-LF group was lower than that of the NA-HF group (*n* = 44, sFer: 44.0 ± 2.8 mg/L vs. *n* = 19, sFer: 65.1 ± 2.8 mg/L, *p* < 0.05) (Table 2).

**TABLE 1:** The baseline characteristics of the NA-HF and A-LF groups.

	NA-HF	A-LF	*p*-Value
Gestational age (week)	35.5 [3.2]	32.1 [3.6]	<0.05*
Birth weight (kg)	2,009.0 [373.2]	1,597.0 [447.3]	<0.05*
Sex [n (%)]			0.589
Male	11 (57.9%)	27 (61.4%)	
Female	8 (42.1%)	17 (38.6%)	
Maternal age (year)	30.5 (0.41%)	30.0 (0.36%)	0.669
Maternal education background [n (%)] ≤ senior middle school	6 (31.6%)	11 (25.0%)	0.292

Values are means ± SEM, n = 19 in NA-HF group/44 in the A-LF group. *p < 0.05 indicates means with significant difference. NA-HF, non-anemia with higher serum ferritin; A-LF, anemia with lower serum ferritin.

**TABLE 2:** Iron status in the NA-HF and A-LF groups.

	NA-HF	A-LF	*p*-Value
**Newborn**			
Hb (g/L)	174.1 [2.8]	114.9 [6.3]	<0.05*
**3 months**			
Hb (g/L)	97.6 [2.7]	102.1 [1.3]	0.127
MCV (fl)	82.1 [1.4]	85.5 [0.9]	0.628
MCHC (g/L)	330.8 [2.8]	330.9 [1.9]	0.550
RDW (%)	13.7 [0.5]	14.5 [0.3]	0.790
SF (μg/L)	65.1 [2.8]	44.0 [2.8]	< 0.05*
**6 months**			
Hb (g/L)	116.6 [2.8]	117.2 [2.5]	0.881
MCV (fL)	79.1 [1.0]	75.5 [2.2]	0.336
MCHC (g/L)	327.1 [3.1]	328.4 [2.4]	0.753
RDW (%)	13.3 [0.4]	13.5 [0.3]	0.655
SF (μg/L)	39.3 [15.0]	30.8 [3.6]	0.460

*Values are means ± SEM, n = 19 in the NA-HF group/44 in the A-LF group.*

*p < 0.05 indicates means with significant difference. Hb, hemoglobin; MCV, mean corpuscular volume; MCHC, mean hemoglobin concentration; RDW, red blood cell distribution width.

**FIGURE 1:**
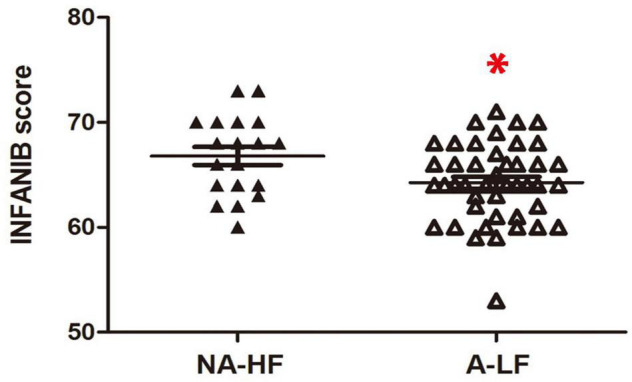
The scores of Infant Neurological International Battery (INFANIB) display a lower value in anemia in the lower ferritin (A-LF) group than non-anemia in the higher ferritin (NA-HF) group at 3 months old. *p < 0.05.

All preterm infants were tested with INFANIB at 3 months old. We found that the overall INFANIB scores were lower in the A-LF group. The discrepancy has statistical significance after controlling by GA and corrected age (*n* = 44, 64.4 ± 0.6 vs. *n* = 19, 66.8 ± 0.9, *p* < 0.05, Figure 1).

### Structural Changes of Neuroimaging in Anemia Preterm Infant With Lower Serum Ferritin

Previous studies have found that structural connections between motor-related brain regions play an important role in movement development (Craig et al., 2020). Only 32 of A-LF infants and 17 of NA-HF infants had qualified MRI examination. MRI quality control was performed as follows: (1) without acquired punctate or focal lesions, marked dilation of the cerebral ventricles on MRI scans; (2) without visible artifacts on MRI scans. The ROI registration files of all the subjects were checked, and the registration effect was good. According to previous studies, a total of 12 connections were examined in the present study, including ipsilateral and contralateral precentral gyrus, cerebellum, thalamus, and striatum. The results showed that there was obvious fiber connection between the ipsilateral and contralateral precentral gyrus–cerebellum, precentral gyrus–striatum, cerebellum–thalamus, and cerebellum–striatum which had significance (Table 3) in both A-LF and NA-HF groups.

**TABLE 3:** The number of fiber connectivity between motor related brain regions.

	NA-HF	A-LF	*p*-value
Precentral gyrus–cerebellum (ipsilateral)	1,572.6 [250.0]	1,751.9 [184.6]	0.568
Precentral gyrus–cerebellum (contralateral)	1,098.7 [175.1]	1,210.4 [143.3]	0.636
Precentral gyrus–striatum (ipsilateral)	1,032.2 [281.4]	900.0 [103.2]	0.664
Precentral gyrus–striatum (contralateral)	6 [1.2]	10.2 [2.1]	0.171
Cerebellum–thalamus (ipsilateral)	109.7 [23.8]	70.4 [6.6]	<0.05*
Cerebellum–thalamus (contralateral)	52.5 [19.6]	42.6 [5.1]	0.628
Cerebellum–striatum (ipsilateral)	94.8 [15.7]	91.1 [14.0]	0.869
Cerebellum–striatum (contralateral)	61.2 [18.3]	56.8 [10.0]	0.821

Values are means ± SEM, n = 17 in NA-HF group/32 in A-LF group. *p < 0.05 indicates means with significant difference.

**FIGURE 2:**
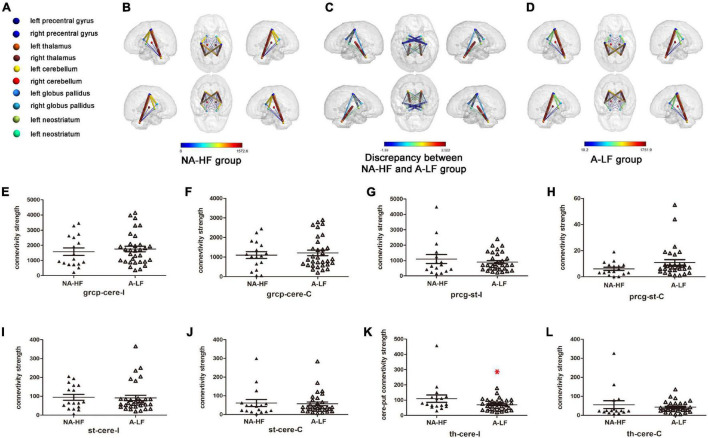
Comparisons of clusters with significant connectivity between motor regions in the A-LF and NA-HF groups with multiple corrections by diffusion tensor imaging (DTI) at 3 months old. **(A)** Color-coded spheres present motor-related ROIs. **(B–D)** Locations and signs of brain structural connectivity between motor-related regions are illustrated. Spheres represent regions of interest (ROIs). Neostriatum and globus pallidus stand for striatum. Sticks with colors from blue to red represent probabilistic brain structural connectivity for an ROI pair. Color bars in **(B,D)** indicate the connectivity enrichment. Color bars in **(C)** indicate the discrepancy between the A-LF group and NA-HF group, respectively. **(E–L)** Comparisons of structural connectivity showed lower enrichment between cerebellum and ipsilateral thalamus in the A-LF group compared with the NA-HF group, and no significant difference was detected between other ROIs. grcp, precentralgyrus; cere, cerebellum; th, thalamus; st, striatum (include caudate nucleus, Cau/globus pallidus, GP); I, ipsilateral; C, contralateral; *p < 0.05.

Then, we compared the above eight connections between the two groups. After controlling GA and physical age, only the connectivity between cerebellum and ipsilateral thalamus in the A-LF group was lower than that in the NA-HF group (109.76 ± 23.8 vs. 70.4 ± 6.6, *p* < 0.05). No statistical difference of the structural connectivity between other ROIs was detected in the two groups (Figure 2).

**FIGURE 3:**
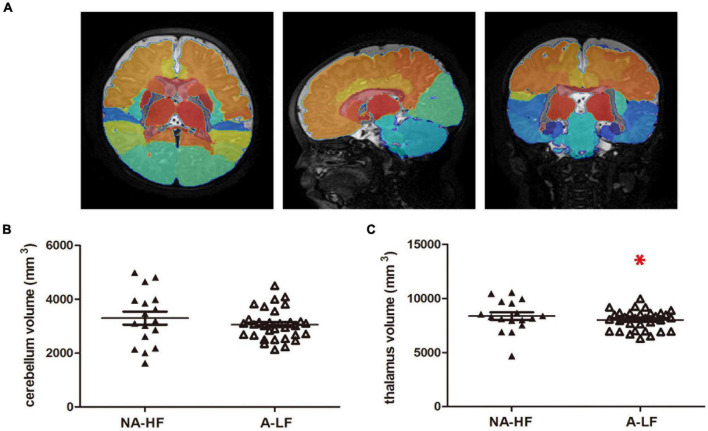
The volumes of motor-related regions. **(A)** The whole brain of each subject was segmented into 83 brain regions using the atlas of the Developing Brain Region Annotation with Expectation-Maximization. **(B)** There was no significant difference in the volumes of cerebellum between the A-LF and NA-HF groups. **(C)** The volume of the thalamus was smaller in the A-LF group than in the NA-HF groups. *p < 0.05.

According to the reported decreased volume of brain regions in IDA infants, the volume of brain region was calculated in the present study. Based on the results of connectivity analysis, we calculated the volume of cerebellum and thalamus. It was found that the overall volume of the left and right thalamus was significantly lower in the A-LF group than that in the NA-HF group (8,369.3 ± 353.2 mm^3^ vs. 6,926.2 ± 552.3 mm^3^, *p* < 0.05), but no significant difference in the cerebellum volume was found between the two groups (Figure 3).

### Iron Status and Scores of Peabody Developmental Motor Scales After Iron Supplementation at 6 Months of Corrected Age in Preterm Infant

To identify the effect of oral iron supplementation, iron metabolism parameters were compared between the two groups at 6 months of corrected age. No difference was detected in Hb (116.6 ± 2.8 g/L, *n* = 19 in the NA-HF group, and 117.2 ± 2.5 g/L, *n* = 44 in the A-LF group) and sFer (39.3 ± 15.0 μg/L, *n* = 19 in the NA-HF group, and 30.8 ± 3.6 μg/L, *n* = 44 in the A-LF group) between the two groups (Table 2).

**FIGURE 4:**
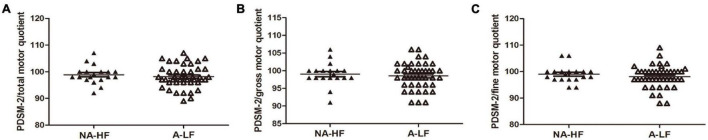
The motor scores accessed by Peabody Developmental Motor Scales, second edition (PDMS-2) after iron supplementation at corrected age of 6 months old. **(A–C)** Although the mean of total motor quotient was slightly lower in A-LF group rather than NA-HF group, there was no significant difference in total motor quotients, gross motor quotients, and fine motor quotients between A-LF and NA-HF groups at this age.

As shown in Figure 4, although the mean of the total motor quotient, gross motor quotient, and fine motor quotient by PDMS-2 assessment was slightly higher in the NA-HF group at 6 months of correct age after 6 months supplementation, there was no significant difference between the two groups (Figure 4).

### Brain Structure–Movement Relationship in Anemia Preterm Infant With Lower Serum Ferritin

**FIGURE 5:**
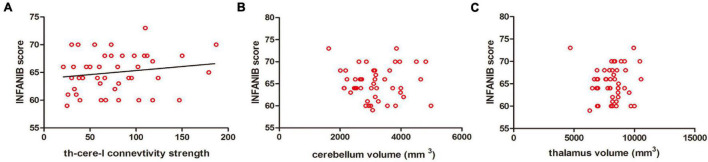
Correlations between the motor scores of INFANIB and brain structural connectivity at 3 months old. **(A)** The structural connectivity between the cerebellum and ipsilateral thalamus was positively correlated with the scores of INFANIB at 3 months old. *r* = 0.347, *p* < 0.05. **(B,C)** The volume of the cerebellum and thalamus were not linearly related with the scores of INFANIB at this age. *r* = 0.056, *p* = 0.930/*r* = 0.047, *p* = 0.951. cere, cerebellum; th, thalamus; I, ipsilateral.

After controlling GA and the age of MRI scan, the structural connection strength between the cerebellum and ipsilateral thalamus was positively correlated with motor scores of INFANIB at 3 months old (*r* = 0.347, *p* < 0.05), while there was no significant correlation between the volumes of cerebellum/thalamus and the motor scores (*r* = 0.056, *p* = 0.930/*r* = 0.047, = 0.951). In addition, we also found that the connection strength was significantly higher in the A-LF group than that in the NA-HF group controlling for the volume of thalamus and cerebellum (*p* < 0.05) (Figure 5).

## Discussion

This study together provides the following evidence: (1) Anemia with lower iron levels during the neonatal period was related to the poor motor performance during early postnatal life among preterm infants. (2) The volume of the thalamus and the structural connection between the cerebellum and ipsilateral thalamus was lower in A-LF than that in NA-HF preterm infants, but only the decreased connectivity between the cerebellum and ipsilateral thalamus in the A-LF group was related with the motor delay. (3) After 6 months of daily iron supplementation, no difference in iron status was detected between the A-LF and NA-HF groups, as well as the motor scores of PDMS-2 assessment.

As premature infants are susceptible to ID than term infants (Moreno-Fernandez et al., 2019), iron detection is necessary at early age of preterm infants. However, considering the limitation of venous blood sampling for iron detection in neonates, as well as the finding that preterm infants with neonatal anemia probably had a lower level of iron than those without neonatal anemia in the previous studies, we analyzed the serum Hb and sFer at 3 months of age. The findings demonstrated that infants with neonatal anemia had lower sFer levels at 3 months old, which may indicate lower iron levels early after birth. Furthermore, previous research found that brain ID occurs earlier than serum ID/IDA (Zamora et al., 2016), and we speculated that lower Hb at birth in the A-LF group might increase the risk of brain ID in our sample.

Many studies have shown that brain iron status is related to neurodevelopment of infants, which is involved in myelination, dopamine neurotransmission, and neuronal metabolism (Beard and Connor, 2003). Most researches focused on ID with cognitive development, but little is known about the motor development of premature infants with ID (Berglund et al., 2013, 2018). In our study, at the same time when blood sampling was tested at 3 months old, we conducted the INFANIB and found that the motor development of A-LF infants obviously lagged behind than that of NA-HF infants. This was consistent with the previous study on term infants that there is poorer motor function in ID group with or without anemia (Shafir et al., 2008).

Currently, most findings showed that brain ID is mainly related to the decrease in the volume of the brain regions (Mudd et al., 2018). Brain regions involved in our study, such as the frontal cortex, striatum, cerebellum, and thalamus, are associated with early motor development (Niendam et al., 2012). As Mudd reported in the pig model, pigs with ID demonstrated reduced iron content in the cerebellum and left cortex as well as decreased gray and white matter compared with the controlled group using the QSM and voxel-based morphometric analysis (Mudd et al., 2018). Another research also indicated that lower iron concentrations in 30-day-old pigs had smaller volume in cortical gray matter in the ID group compared with the control group (Leyshon et al., 2016). In our preterm cohort, the volumes of thalamus were different between the A-LF and NA-HF groups. After further analysis, there is no significant correlation between the volume of thalamus and motor development. It may be related to the limitation of our sample size. Further study with an expanding sample size will be needed to address this issue in more detail.

Despite the volume of brain regions, recent animal studies demonstrated that perinatal ID affected cortical neurons, and both apical and basal dendrites displayed a uniform decrease in branching (Felt et al., 2006; Greminger et al., 2014), which may lead to decreased neuron connection between cortex and other brain regions. Iron is important as it is involved in the production of myelin basic protein (MBP) and maintenance of myelination of neurons in brain gray matter, such as the thalamus (Mills et al., 2010; Huber et al., 2020). Other studies reported that the cerebellum and thalamus are susceptible to ID, which may lead to changes in monoamine metabolism, resulting in functional connection disorder (Felt et al., 2006). Our study has shown that the structural connections between the cerebellum and thalamus were lower in the A-LF than that in the NA-HF groups at 3 months old. The results of regression analysis demonstrated that the level of motor development of infants of the same age was positively correlated with the structural connection between the cerebellum and thalamus. Our study showed that the structural connections between the cerebellum and thalamus in A-LF were obviously lower than that in the NA-HF groups at 3 months old. Moreover, consistent with Andreasen’s findings, the result of our regression analysis demonstrated that the level of motor development of infants at 3 months old was positively correlated with the number of nerve structural connections between the cerebellum and thalamus. ID may lead to the reduction in motor-related neural connections, thus, affecting the level of motor development of premature infants in our cohort. The findings of both studies, one based on probabilistic fiber tracking and another by Andreasen’s team, suggested that anemic infants with lower iron levels had reduced structural connection between the cerebellum and thalamus. This evidence helps us to explain why A-LF preterm infants have poorer motor performance compared with the NA-HF group. Although the locus coeruleus drives disinhibiting in the midline thalamus *via* a dopaminergic mechanism (Beas et al., 2018), the relationship between structural and functional connection remains controversial. Most studies believed that the decrement in structural connectivity is prior to the reduction in functional connectivity, as structural connectivity is the basis of functional connectivity (Hampton et al., 2017), while Betzel et al. (2014) and Bernard et al. (2016) found an increase in functional connection followed by a secondary alteration of structural connection. As previous researches indicated, the findings by Bernard may be related to the reconstruction of synapsis at young age and the compensation of monoamine metabolism (Paolicelli et al., 2011). The findings above support the importance of the detection with DTI to evaluate the network level of structural connection.

To improve the motor development of preterm infants affected by ID, many guidelines for daily iron supplementation were proposed. Although there were discrepancies of the recommended dosage and initiation time of routine iron supplementation on preterm infants among the following consultations, the overall consensus is reached that early iron supplementation can be beneficial to preterm infants. The American Academy of Pediatrics recommends that breast-fed and formula-fed premature infants should receive 2 and 1 mg/kg/day element iron, respectively, from the age of 1–12 months, and the European Society of Pediatric Gastroenterology and Nutrition and the Canadian Pediatric Society recommended a larger dose (Nutrition Committee, Canadian Paediatric Society, 1995; Rao and Georgieff, 2009). ID/IDA during pregnancy was associated with poorer motor development of the offspring, but iron supplementation during pregnancy has little effect on the impairment of motor function (Hernandez-Martinez et al., 2011; Tran et al., 2014). The optional time window of iron supplementation on neurodevelopment remains controversial. A longitudinal study from Costa Rica demonstrated that despite iron therapy in infancy, the motor development of infants with chronic ID was not improved over time (Shafir et al., 2006). However, a randomized controlled trial from Hebei of China demonstrated that iron supplementation during early infancy reduced the proportion of children in the lowest quartile of the locomotor subscale in the child, regardless of whether their mothers were receiving iron supplementation or not during pregnancy (Angulo-Barroso et al., 2016). Similar findings were also reported in another Chinese RCT cohort that iron supplementation from 6 weeks to 9 months had a positive effect on overall gross motor development at 9 months in term infants. These studies indicated the importance of iron supplementation in early infancy (Zhang et al., 2019). However, all of these studies only focused on term infants. Our study added the evidence of the significance of iron supplementation for premature infants that, similar to term infants, daily iron supplementation from early infancy can reverse the delayed motor development of preterm infants. However, whether we can maintain a more desirable iron level of preterm infants and improve their motor ability by long-term regular iron supplementation needs to be evaluated in the future.

Our study had some limitations and are as follows: First, the study was limited by a relatively small sample size, and there is a sample bias between the NA-HF and A-LF groups. It may lead to a statistical bias. The sample size should be enlarged and balanced in future research. Second, considering ethical issues, we did not set up sub-groups without routine iron supplementation. A further well-designed study will help to better monitor the long-term effect of routine iron supplementation among preterm infants and to determine if our results can be generalized. Third, MRI scan including sequence for quantitative susceptibility mapping (QSM) as an indirect quantitative marker of brain iron and one more DTI detection at 6 months old of correct age will be needed to confirm the mechanism that the iron status of preterm infants is related to motor development *via* the decreased brain structural connectivity.

As a conclusion, in this study, we demonstrated that iron status of preterm infants is related to motor development, which is also related with the decreased connectivity between the cerebellum and ipsilateral thalamus. These neuroimaging outcomes together with the infantile iron status and motor abilities in our study provided evidence that structural connectivity assessed by diffusion MRI may serve as a biomarker to predict the motor development in ID preterm infants. Daily iron supplementation at an early age can reverse the delayed motor development in preterm infants with lower iron status.

## Data Availability Statement

The raw data supporting the conclusions of this article will be made available by the authors, without undue reservation.

## Ethics Statement

The studies involving human participants were reviewed and approved by the Ethics Committee of the Children’s Hospital, Zhejiang University School of Medicine. Written informed consent to participate in this study was provided by the participants’ legal guardian/next of kin, for the publication of any potentially identifiable images or data included in this article.

## Author Contributions

ML: conceptualization and methodology. WX, WC, YL, and QZ: investigation and data curation. YL: writing and original draft preparation. TL, FG, and YY: visualization. CJ and JS: supervision, reviewing, and editing the manuscript. All authors contributed to the article and approved the submitted version.

## Conflict of Interest

The authors declare that the research was conducted in the absence of any commercial or financial relationships that could be construed as a potential conflict of interest.

## Publisher’s Note

All claims expressed in this article are solely those of the authors and do not necessarily represent those of their affiliated organizations, or those of the publisher, the editors and the reviewers. Any product that may be evaluated in this article, or claim that may be made by its manufacturer, is not guaranteed or endorsed by the publisher.

## Footnotes

1
www.mrtrix.org
